# The cytoplasmic domain of N-cadherin modulates MMP-9 induction in oral squamous carcinoma cells

**DOI:** 10.3892/ijo.2014.2549

**Published:** 2014-07-21

**Authors:** ANDREW WALKER, RHET FREI, KATHRYN R. LAWSON

**Affiliations:** 1 Department of Biochemistry, Midwestern University, Glendale, AZ; 2 Department of Oral Biology, Eppley Cancer Center, University of Nebraska Medical Center, Omaha, NE, USA

**Keywords:** N-cadherin, matrix metalloproteinase-9, invasion, motility, p120, β-catenin, oral squamous cell carcinoma

## Abstract

Oral squamous carcinoma is the sixth most common cancer worldwide, and one of the most common cancers in developing countries. Regional and distant metastases comprise the majority of cases at initial diagnosis and correlate with poor patient outcomes. Oral epithelia is one of many tissue types to exhibit a cadherin switch during tumor progression, in which endogenous cell adhesion proteins, such as E-cadherin, give way to those of mesenchymal origin. The mesenchymal cell adhesion protein N-cadherin is found at the invading front of oral squamous carcinomas and has been strongly correlated with poor patient prognosis. The goal of the present study was to elucidate the mechanism by which N-cadherin may increase extracellular matrix-associated proteolytic activity to facilitate invasiveness in oral tumor development. The overexpression of N-cadherin in two oral squamous carcinoma cell lines increased motility, invasive capacity and synthesis of matrix metalloproteinase-9 (MMP-9) in a manner that was independent of E-cadherin downregulation. The use of EN and NE chimeric cadherin molecules with reciprocally substituted cytoplasmic domains revealed that optimal induction of MMP-9 synthesis required the cytoplasmic region, but not the extracellular region, of N-cadherin. Utilizing an N-cadherin mutant with impaired p120 binding ability, we found that such mutation resulted in a 4-fold decrease in motility compared to wild-type N-cadherin, but did not affect either MMP-9 expression or motility-normalized invasion. Overexpression of wild-type N-cadherin produced a 27-fold increase in the transcriptional activity of β-catenin, concomitant with increases in MMP-9 transcription. These results suggest that N-cadherin may promote motility and invasiveness through distinct mechanisms, and that β-catenin may be an integral mediator of N-cadherin-dependent invasive signaling in oral epithelia.

## Introduction

Oral cancer is the sixth most common cancer worldwide, and is responsible for 128,000 deaths each year (1). In south-central Asian countries, where tobacco use is prevalent, oral cancer is the most common cancer in men, contributing up to 25% of all new cases of cancer (2). Delayed patient presentation combined with lack of effective modalities of treatment of advanced oral cancers contribute to a 5-year survival rate of 50% (2) a figure that has changed little over the last 40 years (3). In the United States, the 5-year survival rate for patients with localized oral tumors is roughly 80%, however, nearly two-thirds of patients diagnosed with oral cancer initially present with regional or distant metastases, which are correlated with survival rates of 57 and 37%, respectively (3). Because invasion is a key correlate of patient survival, a more definitive understanding of the specific mechanisms associated with oral squamous carcinoma metastasis is critical to improving patient outcomes in both preventive and therapeutic contexts.

Tumor metastasis is facilitated by a highly coordinated tandem of increased migratory ability coupled with increased proteolytic activity towards extracellular matrix components. The acquisition of such characteristics is one of the hallmark features of a cellular dedifferentiation program termed epithelial-to-mesenchymal transition (EMT) (4). An early event in EMT is the downregulation of E-cadherin, a transmembrane glycoprotein that plays a critical role in epithelial cell adhesion and in the maintenance of the polarity of the epithelial layer. Loosening of cell-cell contacts is part of a coordinated alteration of the epithelial phenotype that results in the acquisition of mesenchymal characteristics: loss of polarity, increased motility, fibroblastic morphology and expression of mesenchymal proteins such as vimentin. It is this mesenchymal reprogramming that enables cells to dissociate from the primary tumor nest (5,6) and eventually invade into the surrounding tissue.

A well-documented phenomena of EMT is a ‘cadherin switch’, in which E-cadherin loss is accompanied by the *de novo* expression of the mesenchymal adhesion protein N-cadherin (7,8). A growing body of research has identified a role for N-cadherin in tumor progression that is causative rather than coincidental. Ectopic expression of N-cadherin in oral, breast and bladder carcinoma cell lines has been shown to increase both motility and invasiveness (9–11). *De novo* expression of N-cadherin has been found in both poorly-differentiated tumors and the invasive front of well-differentiated tumors in several tissue types (12–14). In oral squamous carcinomas, the presence of N-cadherin has been strongly correlated with loco-regional invasion and poor patient prognosis (14,15).

Invasion is facilitated by both increased migration and by increased activity of matrix metalloproteinases, a family of zinc-dependent endopeptidases that degrade extracellular matrix components (16). In several cohort studies of oral squamous carcinoma, elevated expression of matrix metalloproteinase-9 (MMP-9) was correlated with regional lymph node and/or distant metastases (17,18) and adversely correlated with survival (17). MMP-9 has been identified as a modulatory target of both E- and N-cadherin-dependent signaling (11,19–21). In oral keratinocytes and bronchial cells, MMP-9 expression was suppressed by E-cadherin-mediated adhesion (20–22), whereas in breast cells, MMP-9 expression increased in the presence of N-cadherin (11,19).

Although the role for N-cadherin in conferring migratory ability to epithelial cells is well established (9,10,23,24), very few studies have examined the effect of ectopic N-cadherin expression on matrix metalloproteinase activity. In breast cells, N-cadherin expression potentiated the MMP-9 expression that resulted from fibroblast growth factor receptor (FGFR) signaling, but did not increase basal MMP-9 expression in untreated cells (19). The means by which N-cadherin promotes invasion may be tissue-specific, however, as a similar response to FGF was not seen in N-cadherin expressing bladder cancer cells (11). Oral squamous cells are one of many cell types in which the presence of N-cadherin decreases E-cadherin protein levels (10), thus raising the possibility that oral tumor progression is facilitated not only by *de novo* N-cadherin signaling but also by concomitant decreases in E-cadherin function.

In the present study, we utilized two oral squamous carcinoma cell lines to examine the relative roles of N- and E-cadherin in promoting matrix metalloproteinase expression and invasive signaling in oral cancer. We also utilized chimeric constructs consisting of reciprocally substituted E- and N-cadherin domains to identify features of N-cadherin that are essential for matrix metalloproteinase expression, migration and invasion in oral squamous cells. Finally, we have determined the relevance of the cadherin-associated proteins and transcriptional modulators β-catenin and p120 in facilitating N-cadherin-dependent invasion. Our data demonstrate that it is the cytoplasmic portion of N-cadherin which confers increased MMP-9 expression to oral squamous carcinoma cells, and suggest a role for the N-cadherin cytoplasmic binding partner β-catenin in modulating MMP-9 transcription.

## Materials and methods

### Cell culture

The oral squamous carcinoma cell lines Tu167 (25) and SCC1 cells (26) were maintained at 37°C, 5% CO_2_, in minimum essential medium (Sigma) supplemented with penicillin, streptomycin and 10% fetal bovine serum (PAA). Murine fibroblast NIH3T3 cells (American Tissue Culture Collection) were maintained in Dulbecco’s modified Eagle’s medium supplemented with 10% newborn calf serum, penicillin and streptomycin.

### Retroviral transduction

cDNAs encoding full-length human N-cadherin (27), chimeric EN and NE cadherins (27) or p120-uncoupled N-cadherin were subcloned into the retroviral expression vector LZRS-MS-Pac (28). Construction of the chimeric EN and NE cadherins has been previously described (9). The N-cadherin mutant was generated using a QuickChange site-directed mutagenesis kit. This cDNA contains three sequential alanine substitutions (E780A, E781A and D782A) (23,29), which correspond to homologous mutations in the E-cadherin sequence that abrogate binding of p120 (30). For depletion of endogenous cadherin transcripts, oligonucleotides directing the formation of short hairpin RNAs against human N-cadherin (17) or E-cadherin (GGCCTCTACGGTTTCATAA) were cloned into pSuper. retro.puro retroviral expression vectors (Oligoengine). Production of amphotropic retrovirus and subsequent infection of Tu167 and SCC1 cells was performed as previous described (31).

### Immunoblot analysis

Detergent extraction of cell monolayers and SDS-PAGE was performed as described previously (32). The mouse monoclonal antibodies directed against N-cadherin (13A9), E-cadherin (4A2), P-cadherin (6A9), β-catenin (15B8), α-catenin (1G5) have been described previously (31). The mouse monoclonal anti-p120 antibody was purchased from BD Biosciences, and mouse monoclonal anti-β-tubulin from the Developmental Studies Hybridoma Bank (University of Iowa).

### Migration and invasion assays

Tu167 and SCC1 cells were plated in serum free media in 24-well Matrigel-coated or uncoated Boyden chambers (8 μm pores, BD Biosciences) at a cell density of 7.5×10^4^ cells/chamber. Media conditioned by NIH3T3 cells (Dulbecco’s modified Eagle’s medium + 10% newborn calf serum) was used as a chemoattractant in the lower chamber. Membranes were collected after 24 (motility) or 48 (invasion) hours, and the upper surface of each insert membrane was scraped with a cotton swab to remove cells that had not traversed through to the other side. The remaining cells were stained with Diff-Quick (Dade) and counted. Quantitation was performed by counting cells in 9 random fields of view at 100× and expressing the average number of cells/field of view. All experiments were repeated in triplicate. The data are presented as the average of three independent experiments with the standard deviation of the average indicated. For presentation of the Invasion Indices, cell counts for invasion were normalized to the counts obtained for motility of each respective cell line, and expressed as a percentage compared to controls.

### Substrate gel electrophoresis

For zymographic analysis, confluent cell cultures in 6-well plates were incubated in serum-free MEM for 18 h and then in newly replaced collection media (serum-free MEM) for an additional 24 h. To normalize to cell number at time of media collection, cell monolayers were rinsed twice with phosphate-buffered saline and lysed with RIPA buffer. Volumes of conditioned MEM media proportional to the total recovered protein from each culture were analyzed for gelatinase activity according to the protocol of Leber and Balkwill (33). Gels were imaged and areas of clearing (representing gelatinase activity) were quantitated using the Odyssey Infrared Imaging System (Licor).

### Luciferase reporter assays

Cells were transfected with TOPFlash or FOPFlash firefly luciferase vectors, and a *Renilla* luciferase control vector (Promega). Lysates were collected for analysis 24 h after transfection. Firefly and *Renilla* luciferase activities were determined utilizing the Dual Glo Luciferase Assay Kit (Promega). The values reported were corrected for TCF/LEF-independent transcriptional activation and transfection efficiency. Data shown are the average of three independent experiments with standard deviations indicated.

### Real-time PCR

Total RNA was prepared using the High Pure RNA Isolation Kit (Roche). RNA (2 μg) was reverse-transcribed using the High Capacity cDNA Synthesis Kit (Invitrogen). Quantitative gene expression was performed for MMP-9 and glyceraldehyde-3-phosphate dehydrogenase (GAPDH) using the TaqMan Gene Expression system with gene-specific primers and probes (for MMP-9, Hs00234579_m1; for GAPDH, Hs03929097_g1). PCR reactions were performed on a StepOne Plus Real-Time PCR System (Applied Biosystems) using TaqMan Universal PCR master mix according to standard manufacturer’s protocol. The data were then quantitated using the comparative C_t_ method for relative gene expression utilizing GADPH values as an endogenous control.

## Results

### Effect of altered cadherin expression on adherens junction components

**Figure 1:**
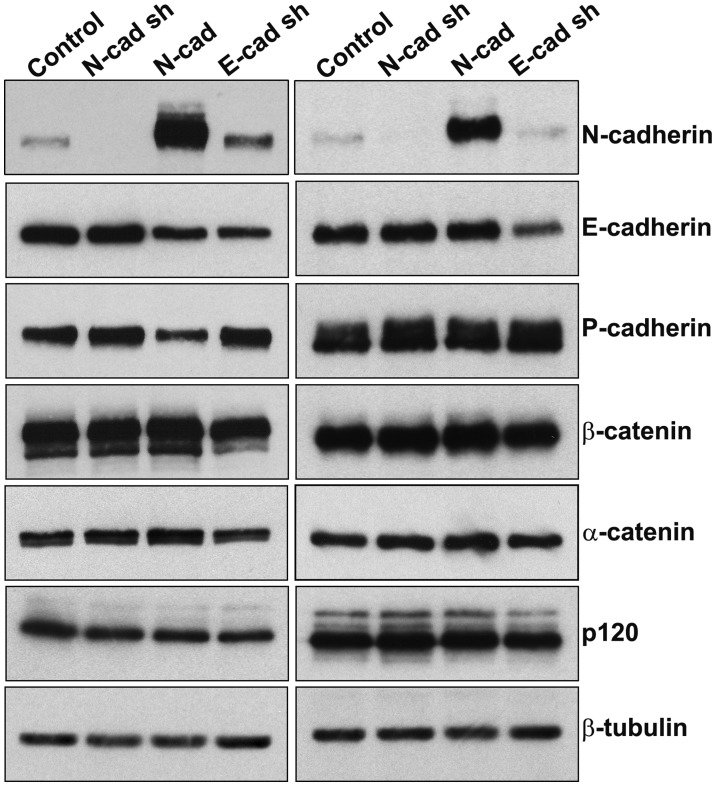
Western blot analysis of N-, E- or P-cadherin, α- or β-catenin, p120ctn and β-tubulin in retrovirally transduced Tu167 (left panel) and SCC1 (right panel) cells. Cells expressed either an N-cadherin cDNA (N-cad) for overexpression, or small hairpin RNAs directed against firefly luciferase (control), human E-cadherin (E-cad sh) or human N-cadherin (N-cad sh).

Oral squamous carcinoma cells are one of many tissue types that downregulate E-cadherin in response to the overexpression of mesenchymal cadherins (31,34). The retention of E-cadherin in invasive, N-cadherin-expressing cells suggests that the loss of E-cadherin function is inconsequential to N-cadherin-mediated invasive signaling (10,11). However, E-cadherin has been shown to be suppressive of invasion in several tissues, including oral epithelia (20–22). Such data raise the possibility that decreased E-cadherin function may independently contribute to increased proteolytic activity. In order to directly define the specific contributions of E-cadherin loss and N-cadherin expression to the invasiveness of oral squamous carcinoma cells, N- or E-cadherin expression was independently altered in two oral squamous carcinoma lines, Tu167 and UM-SCC1 (SCC1). Retrovirally-encoded N-cadherin cDNA or small hairpin RNAs (shRNA) were used to alter N- and E-cadherin levels in both parental cell lines. Endogenous N-cadherin levels in each parental cell line are relatively low en masse (Fig. 1A), but an immunofluorescence analysis of these cells revealed a marked heterogeneity of N-cadherin expression (data not shown). To reduce N-cadherin expression across the entire cell population, an shRNA vector against N-cadherin was used to deplete N-cadherin from the entire population.

As shown in Fig. 1, in Tu167 cells, overexpression of N-cadherin decreased, but did not abolish, expression of epithelial E- and P-cadherins. In SCC1 cells, N-cadherin overexpression did not alter levels of E- or P-cadherin. This decrease in E-cadherin did not affect levels of N-cadherin expression compared to controls. Levels of β-catenin, α-catenin, and the isoforms/levels of p120 catenin were unaffected by the various expression constructs. Most importantly for the purposes of these studies, shRNA-mediated E-cadherin depletion was either similar (Tu167) or more extensive (SCC1) than the E-cadherin depletion seen as a result of N-cadherin overexpression. Thus, the invasive effects of E-cadherin depletion may be investigated independently of the consequential E-cadherin depletion caused by N-cadherin overexpression.

### Effect of altered cadherin expression on colony morphology of oral squamous carcinoma cells

**Figure 2:**
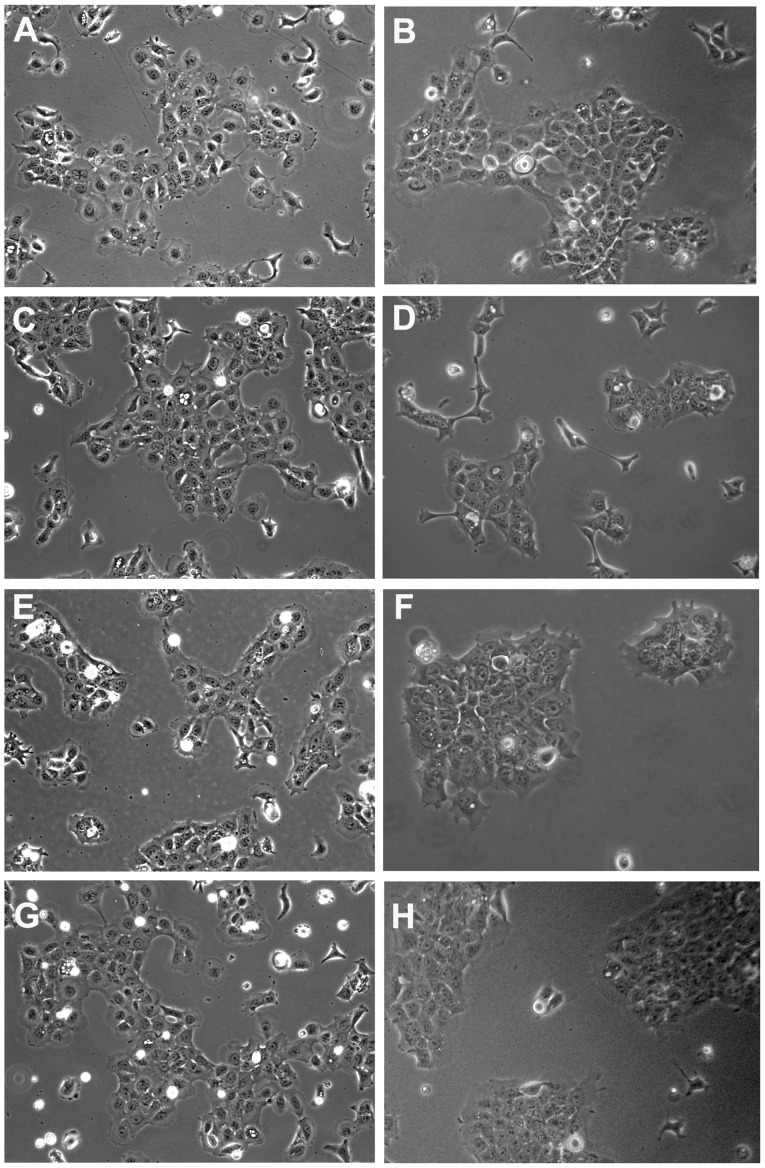
Morphological analysis of TU167 and SCC1 cells expressing varied levels of N- and E-cadherin. Cells utilized for the western blot data in Fig. 1 were photographed 24 h after plating using a 10× objective. Shown are (A, C, E and G) Tu167 cells and (B, D, F and H) SCC1 cells stably transduced with (A and B) a control vector or vectors encoding (C and D) an N-cadherin shRNA, (E and F) N-cadherin cDNA or (G and H) E-cadherin shRNA.

In prior studies utilizing a dexamethasone-inducible N-cadherin cDNA, SCC1 cells exhibited a scattered phenotype in response to increased expression of N-cadherin protein (34). Similar studies which utilized a constitutively-expressed N-cadherin in breast cells showed no effect on cell or colony morphology (24). In the present study, neither the constitutive expression of N-cadherin (Fig. 2E and F) nor decreased expression of E-cadherin (Fig. 2G and H) resulted in cell scattering. In fact, N-cadherin overexpression increased colony formation in both cell lines, resulting in fewer scattered cells within each culture (compare Fig. 2A and B to E and F). E-cadherin depletion had no effect on cell scattering (Fig. 2G and H).

### N-cadherin expression, but not E-cadherin depletion, increases MMP-9 activity

**Figure 3:**
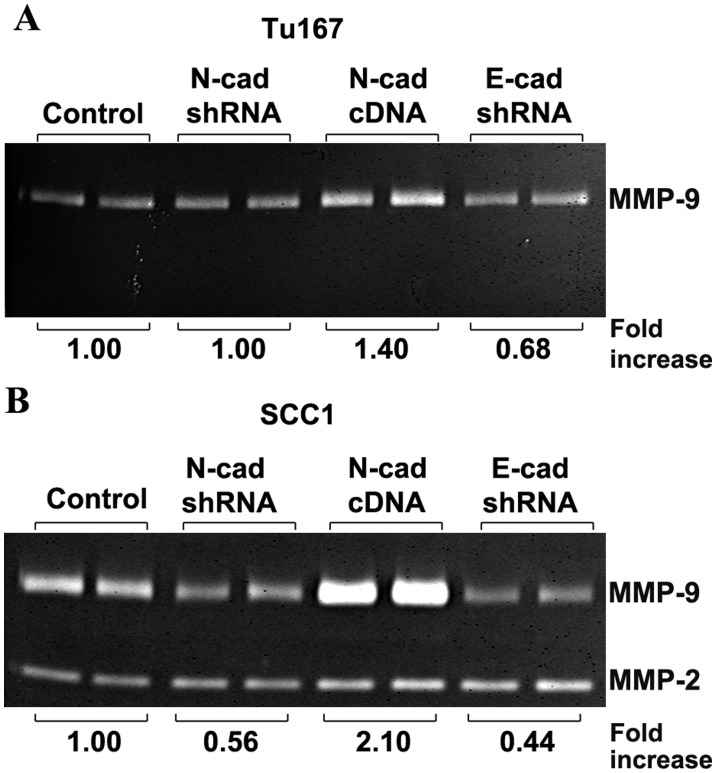
Zymographic analyses of MMP-2 and MMP-9 in transduced cells. (A) Control Tu167 and (B) SCC1 cells and cells containing either N-cadherin cDNA or N- and E-cadherin shRNAs were plated in duplicate. Cells were exposed to serum-free media for 24 h, at which time media from each plating was independently collected and subject to gelatin-embedded SDS PAGE and zymography. Areas of gelatinolytic activity were quantified, and are reported as the average fold-change of each sample compared to that of the respective control value for each cell line.

Gelatin zymography of conditioned serum-free media (Fig. 3) was performed to quantitate MMP secretion from control cells, cells with depleted N-cadherin (N-cad shRNA), overexpressed N-cadherin (N-cad cDNA), and depleted E-cadherin (E-cad shRNA). In both Tu167 and SCC1 cell lines, the overexpression of N-cadherin increased MMP-9 activity 1.4-fold and 2-fold, respectively. The depletion of E-cadherin did not increase MMP-9 activity in either cell line, and in fact resulted in a moderate suppression of MMP-9 activity (Fig. 3A and B). In SCC1 cells (Fig. 3B), depletion of N-cadherin by shRNA resulted in a moderate decrease in MMP-9 secretion compared to control. SCC1 cells also secreted MMP2, which was unaffected by perturbations in N- or E-cadherin. Tu167 cells did not express MMP-2 under any conditions analyzed.

### N-cadherin expression increases invasion in a migration-dependent and -independent manner

**Figure 4:**
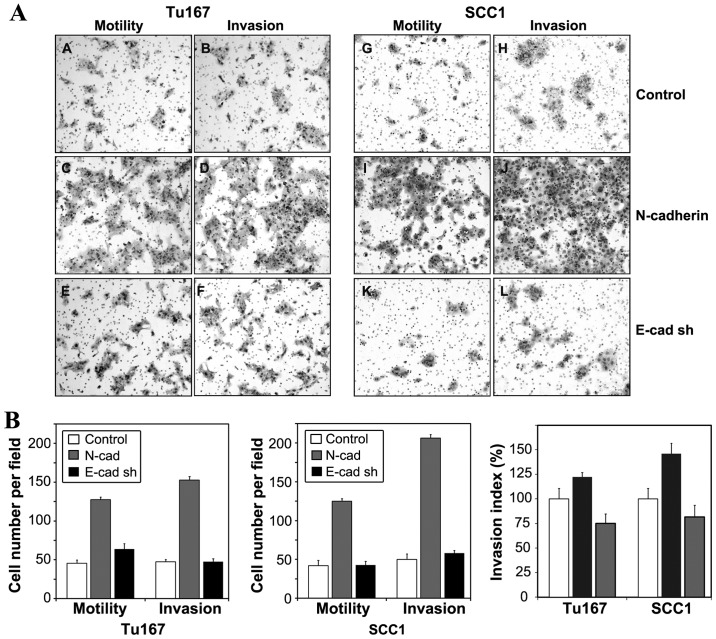
*In vitro* migration and invasion assays of oral squamous carcinoma cells. Retrovirally-infected Tu167 and SCC1 oral squamous cells were plated on uncoated and matrigel-coated Boyden chambers and allowed to migrate towards fibroblast-conditioned chemoattractant media for 24 or 48 h. (A) Representative photographs of control and matrigel-coated filters with migrated cells expressing control vector, N-cadherin cDNA or E-cadherin shRNA. (B) Quantitation of migration and invasion for each cell line. Each cell line independently expressed a control vector (white bars), N-cadherin cDNA (grey bars) or E-cadherin shRNA (black bars). Motility and invasion are represented as cells per field. The invasion index is reflected as the quantity of cells traversing matrigel-coated filters normalized to respective values from uncoated filters to account for alterations in cell migratory ability.

The effect of altered N- and E-cadherin expression on both cell motility and invasion was examined by Transwell migration assay (Fig. 4A and B). Representative images of cells which traversed membrane filters are shown in Fig. 4A. Quantitation of cell migration data (Fig. 4B) revealed increases in motility and invasion in N-cadherin-expressing cells, but not in E-cadherin depleted cells. To eliminate the contribution of increased cell motility to the invasion data, invasion data were normalized to migratory data to generate an Invasion Index (Fig. 4C), which more accurately depicts the proteolytic contribution of N-cadherin to cell invasion. The Invasion Indices demonstrated that overexpression of N-cadherin increased proteolytic activity in Tu167 and SCC1 cells. The invasive capacity of E-cadherin-depleted cells was minimal. These findings are consistent with the relative increases in MMP-9 synthesis demonstrated by zymography (Fig. 3). SCC1 cells displayed a more robust increase in MMP-9 expression than Tu167 (Fig. 3), and displayed a correspondingly greater increase in invasive capacity (Fig. 4C).

### The cytoplasmic region of N-cadherin is required for induction of MMP-9 expression

**Figure 5:**
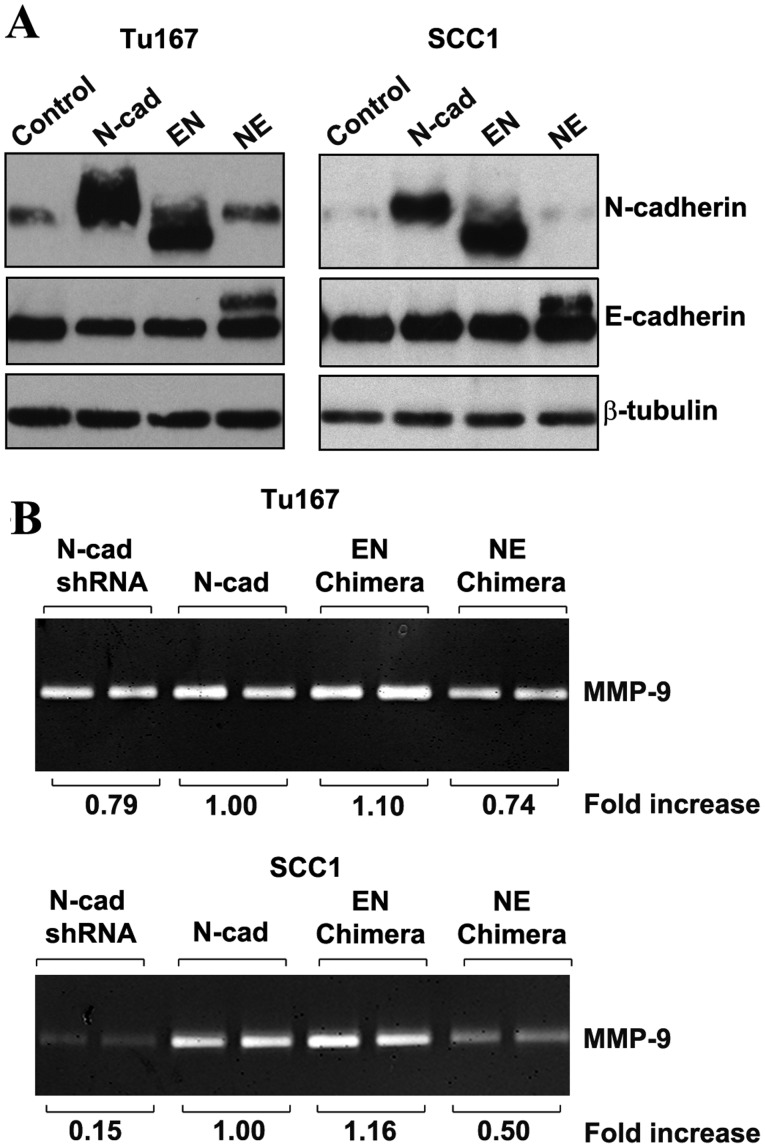
(A) Western blot analysis of oral squamous cells retrovirally transduced with the EN and NE chimeric cadherin constructs. Lysates from cells expressing a control vector, full-length N-cadherin (N-cad) or the EN (EN chimera) and NE (NE chimera) chimeras were analyzed by western blot analysis, utilizing N- and E-cadherin antibodies that recognize an epitope located in the cytoplasmic region of each cadherin. (B) Zymographic analysis of cells overexpressing chimeric cadherin molecules. Duplicate, independently prepared conditioned media were analyzed by gelatinolytic zymography. Numbers represent the average fold-change of each sample compared to that of the respective N-cadherin value for each cell line.

An oral squamous carcinoma cell model has previously been utilized to identify the fourth extracellular repeat domain (EC4) of N-cadherin as the region responsible for conferring increased motility (9). In breast cells, this same region of N-cadherin mediated increased MMP-9 synthesis in an FGF2-dependent manner (19). Because N-cadherin overexpression increased both MMP-9 and invasion in oral squamous cells (Figs. 3 and 4), studies utilizing chimeric cadherin constructs were undertaken to better define the region(s) of N-cadherin that may mediate invasive signaling. Oral squamous cells were independently transduced with retroviral vectors that coded for chimeric cadherin molecules. The EN chimera consisted of the extracellular and transmembrane domains of E-cadherin, and the cytoplasmic portion of N-cadherin. The NE chimera consisted of the extracellular and transmembrane regions of N-cadherin, and the cytoplasmic portion of E-cadherin. Western blot analysis (Fig. 5A) was performed to confirm expression of chimeric proteins in transduced cells. Antibodies directed against cytoplasmic epitopes of E- and N-cadherin were utilized to verify overexpression of chimeric molecules (Fig. 5A).

The EN chimera (EN), which lacked the N-cadherin extracellular domain, retained the ability to induce MMP-9 in both Tu167 and SCC1 cells (Fig. 5B). When the cytoplasmic domain of N-cadherin was lacking (i.e., the NE chimera), MMP-9 levels were reduced to control levels in Tu167 cells and markedly decreased in SCC1 cells (Fig. 5B). These results suggest that invasive signaling is conferred by the cytoplasmic domain of N-cadherin, and that, in oral squamous cells, the N-cadherin EC4 domain is dispensable for increased MMP-9 synthesis.

### P120 is necessary for N-cadherin-induced motility, but not invasion

**Figure 6:**
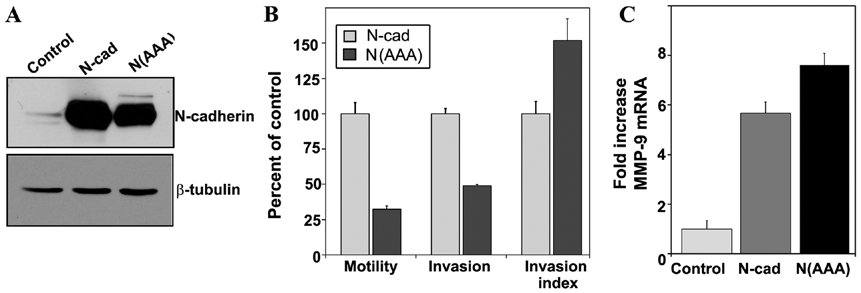
(A) Western blot analysis of SCC1 cells overexpressing either wild-type N-cadherin protein (N-cad) or the N(AAA) mutant, which was designed to impair binding to p120 catenin. Antibodies were directed against the cytoplasmic domain of N-cadherin or a β-tubulin loading control. (B) *In vitro* migration and invasion assays of wild-type N-cadherin and N(AAA)-expressing SCC1 cell lines. Motility and invasion are presented as cells per field. Invasion index as the number of invading cells from each cell line normalized to their respective values from the corresponding motility assay. (C) Relative levels of MMP-9 transcript levels in cells expressing control, N-cadherin and N(AAA) constructs, as determined by real-time PCR. Data are presented as fold-increase compared to control normalized to a GAPDH internal standard.

The cytoplasmic region of N-cadherin interacts with two proteins that are known to play key roles in tumor progression: p120 catenin and β-catenin (35,36). P120 has previously been shown to be integral to the ability of ectopically expressed mesenchymal cadherins to increase motility and, by consequence, invasiveness of epithelial cells (23). This phenomenon is dependent upon proper binding of p120 to the cadherin juxtamembrane domain (23,29,30). To determine the relevance of such interactions with respect to MMP-9 expression, SCC1 cells were transduced with a mutated version of N-cadherin that contained three proximal alanine substitution mutations in the p120 binding domains N(AAA). For both E- and N-cadherin (29,30) homologous mutations have been shown to abrogate binding of each cadherin to p120, and functionally interfere with p120-dependent activities (23). Western blot analysis was used to confirm expression of the mutated N-cadherin protein (Fig. 6A).

Consistent with previous studies (23,29) cells expressing p120-uncoupled N-cadherin exhibited a four-fold decrease in motility compared to cells with wild-type N-cadherin, which corresponded to a decrease in invasion (Fig. 6B). However, the Invasion Index value for cells expressing the N(AAA) mutant demonstrated a 50% increase in invasive capacity compared to control cells (Fig. 6B). An analysis of MMP-9 transcript levels in these cells also revealed no impairment in MMP-9 expression as a result of the N(AAA) mutation (Fig. 6C). These data suggest that although p120 may be critical for N-cadherin mediated motility in oral squamous carcinoma cells, it does not play a role in the proteolytic aspect of invasion.

### N-cadherin overexpression increases transcriptional activation of β-catenin target genes

**Figure 7:**
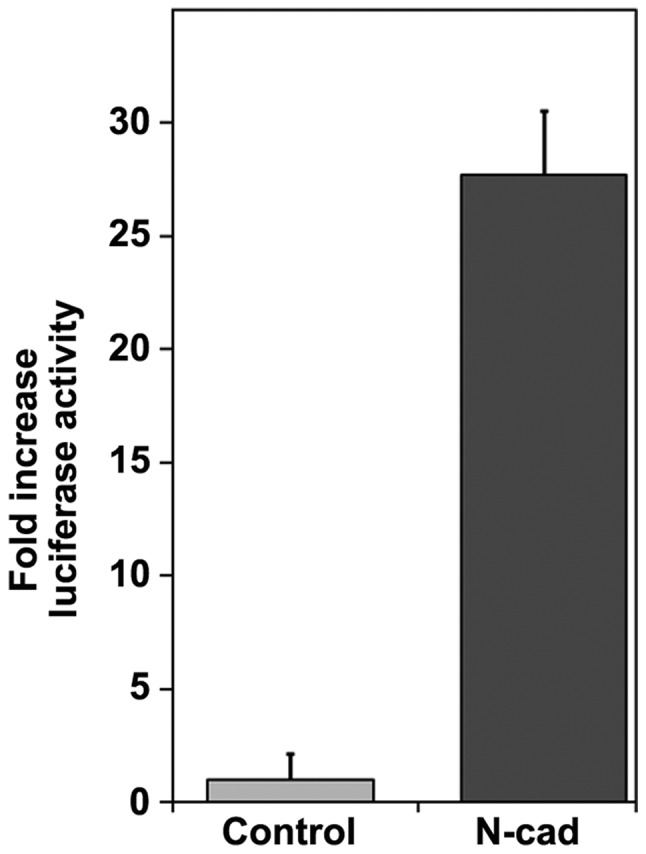
Transcriptional activity of β-catenin as measured by TOPFlash luciferase reporter assay. Control and N-cadherin-overexpressing cells were simultaneously transfected with either a pTOPFlash or pFOPFlash firefly luciferase reporter or a constitutively expressed pRL *Renilla* luciferase vector as an internal control. Luciferase activity was measured 24 h after transfection. Values for each line were first normalized with regard to both FOPFlash activity and *Renilla* luciferase activity. Data are presented as fold-increase compared to control.

One of the most well characterized interactions of the cytoplasmic portion of N-cadherin is with that of the protein β-catenin. β-catenin has been shown to function as a modulator of both cytoskeletal attachment and TCF/LEF-dependent transcription (37,38). β-catenin has also been shown to increased transcription of several matrix-metalloproteinase genes, including MMP-9 (39,40). Overexpression of wild-type N-cadherin increased β-catenin-dependent transcriptional activation of a luciferase reporter by 27-fold compared to control cells (Fig. 7).

## Discussion

The present study demonstrated that in oral squamous cells, the overexpression of N-cadherin, but not loss of E-cadherin, is sufficient to increase levels of MMP-9 transcript and protein levels (Figs. 3 and 6C). N-cadherin expression also increased invasion in a manner that was not entirely the result of increased migratory capacity. These data are in contrast to studies by Suyama *et al*, who demonstrated robust N-cadherin-dependent increases in MMP-9 only upon administration of fibroblast growth factor (19). In our hands, FGF was not necessary for increased levels of MMP-9 in oral squamous cells, nor did treatment of these cells with FGF produce further increases in MMP-9 gelatinase activity (unpublished data). Additional studies performed in breast cells have demonstrated that N-cadherin expression stimulates both invasion and motility to relatively the same degree compared to control cells (10), further supporting the notion that N-cadherin expression in breast cells drives motility but may play less of a role in modulating proteolytic enzyme expression than it does in oral squamous carcinoma. The mechanism by which N-cadherin increases MMP-9 expression and invasion in oral squamous carcinoma cells appears to be a function of N-cadherin that is independent of E-cadherin downregulation. The shRNA-mediated decreases in E-cadherin were not able to substitute for N-cadherin overexpression with regard to increasing invasive characteristics (Figs. 3 and 4).

Because the extracellular domains of N-cadherin did not appear to play a role in MMP-9 induction (Fig. 5), we turned our investigation to cytoplasmic N-cadherin binding partners that have previously been shown to mediate aggressive signaling. P120 catenin interacts with the N-cadherin juxtamembrane region and facilitates lateral clustering of surface cadherins to provide strength to the adherens junction (41). P120 has also been shown to act as a critical modulator of N-cadherin-dependent invasion and migration through its activation of Rho family GTPases (23). Consistent with other studies demonstrating the critical role of p120 in mesenchymal-cadherin-induced motility, migration conferred by the N(AAA) N-cadherin mutant, which is known to be deficient in binding to p120, was markedly reduced compared to controls. However, cells expressing the N(AAA) mutant exhibited an invasive capacity and MMP-9 expression level that was even greater than that seen with expression of wild-type N-cadherin (Figs. 6B and 7). These data suggest that the p120-binding domain of N-cadherin was not a critical element in the induction of MMP-9.

A second binding partner of the cytoplasmic portion of N-cadherin is β-catenin, a growth-associated protein that is dually resident within the adherens junction and in the nucleus, where it functions as a coactivator of the Tcf/Lef family of transcription factors (38). In the present study, N-cadherin overexpression increased β-catenin-dependent transcriptional activity 27-fold (Fig. 7D) in the absence of increased β-catenin synthesis (Fig. 1A). MMP-9 transcripts were also increased in cells expressing either wild-type N-cadherin or the mutant N(AAA) cadherin (Fig. 6C), which is known to retain functional binding of β-catenin (30). Previous studies suggest a high likelihood that the increased MMP-9 activity in N-cadherin overexpressing cells is due to increased β-catenin transcriptional activity. The promoter region of MMP-9 contains Tcf/Lef consensus sequences (39,40), and β-catenin transcriptional activity has been positively correlated with increased MMP-9 transcript levels in several cell types, including oral squamous cells (39,42).

The N-cadherin-dependent increase in β-catenin transcriptional activation is in discordance with previously proposed hypotheses that the sequestration of β-catenin within adherens junctions interfered with the ability of β-catenin to function as a transcriptional activator (43,44). Such a mechanism is unlikely in oral squamous carcinoma cells, however, as depletion of E-cadherin did not increase invasive capacity (Fig. 4B) or synthesis of MMP-9 (Fig. 3), and overexpression of N-cadherin potentiated both MMP-9 synthesis (Fig. 3) and β-catenin-dependent transcriptional activity (Fig. 7).

How then might N-cadherin overexpression positively regulate the nuclear activities of β-catenin? Recent research suggests that the initial association of β-catenin with cadherins may be vital to the ability of β-catenin to function as a transcriptional activator. In MDCK cells, the association of β-catenin with E-cadherin and the internalization of membrane-resident E-cadherin complexes were necessary to maintain β-catenin-mediated transcriptional activation of reporter genes (45). This mechanism may be applicable to overexpression of N-cadherin as well, as the depletion of N-cadherin in N-cadherin-expressing HEK293 cells and in murine embryos significantly decreased β-catenin-mediated transcriptional activity (45). Howard *et al* (45) have proposed a model whereby β-catenin is rendered transcriptionally competent as the result of its adhesion to cadherin molecules at the adherens junction. The subsequent internalization of membrane-resident cadherins allows dissociation of this ‘primed’ form of β-catenin, enabling transcriptional activation of Tcf/Lef target sequences. It is worth noting that distinct pools of adhesive versus transcriptionally active β-catenin have been structurally characterized (44). Recent studies in neural cells suggest that N-cadherin may play a pivotal role in altering the balance of these pools by promoting the phosphorylation of β-catenin by the AKT protein kinase (46).

The present study demonstrates distinct roles of N-cadherin in promoting motility and invasion-related proteolysis. Although p120 is critical for increased motility, p120 does not appear to function in the induction of matrix metalloproteinases. Our data suggest that increased transcriptional activation of matrix metalloproteinases, and increased invasive capacity, was instead mediated by increased β-catenin transcriptional activity. Such regulation of β-catenin may have implications beyond increased matrix metalloproteinase activity, as β-catenin-dependent transcription is integral to a number of signaling pathways essential to tumor progression (36,37). Further studies will be necessary to elucidate the mechanisms by which N-cadherin-dependent increases in β-catenin function may modulate tumor invasion in oral squamous carcinoma cells.
